# School Closures and Student Contact Patterns

**DOI:** 10.3201/eid1702.100458

**Published:** 2011-02

**Authors:** Charlotte Jackson, Punam Mangtani, Emilia Vynnycky, Katherine Fielding, Aileen Kitching, Huda Mohamed, Anita Roche, Helen Maguire

**Affiliations:** Author affiliations: London School of Hygiene and Tropical Medicine, London, UK (C. Jackson, P. Mangtani, K. Fielding);; Health Protection Agency, London (E. Vynnycky, A. Kitching, A. Roche, H. Maguire);; Health Protection Agency, Birmingham, UK (H. Mohamed)

**Keywords:** Disease outbreaks, influenza, human, social behavior, schools, Great Britain, school closures, viruses, expedite, dispatch

## Abstract

To determine how school closure for pandemic (H1N1) 2009 affected students’ contact patterns, we conducted a retrospective questionnaire survey at a UK school 2 weeks after the school reopened. School closure was associated with a 65% reduction in the mean total number of contacts for each student.

During pandemic (H1N1) 2009, several countries closed schools (*1**–**6*) to slow virus transmission. The effects of such school closures on student contact patterns have not been directly quantified. We report these effects for students from a UK secondary school.

## The Study

We retrospectively surveyed 128 students at a coeducational, state secondary school in an urban area of West Midlands, UK, where attack rates for pandemic (H1N1) 2009 were high and (as of March 2010) levels of unemployment were among the highest in Great Britain (*7*). The head teacher selected 1 class from each of years 7–10 (equivalent to US grades 6–9, student ages 11–15 years) to participate. The school had closed for 1 week in mid-June 2009, reopened for 2 days, then closed for another week. Questionnaires were completed during class ≈2 weeks after the school reopened the second time. An electronic version of a similar questionnaire pilot tested at another school had been found comprehensible and acceptable to participants. The London School of Hygiene and Tropical Medicine ethics committee approved the study; the Health Protection Agency approved it as part of wider outbreak investigations not requiring additional approval.

Students reported how many times they visited specified public places before the school closure and how many times they visited these places during closure (children had been advised to not visit public places only if they were symptomatic). Students also provided information about persons who looked after them during closure.

For typical school days before and during closure, students reported the number of different persons spoken to (contacted) in the following groups: contacts who attended their school (contacts from the same class [classmates], the same year but a different class [yearmates], and the same school but a different year [schoolmates]) and others (age stratified to reflect the UK school system). Students were asked whether they were ill during closure and whether being ill affected their contact patterns.

**Figure 1:**
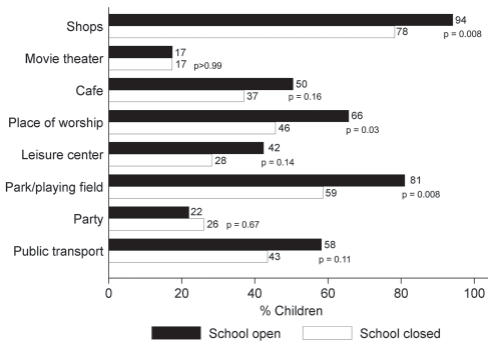
Visits to public places during open and closure periods of a UK secondary school, June–July 2009. Percentage of students visiting public places >1×/week while the school was open (n = 99–103, depending on the place) and while it was closed (n = 46). Numbers after bars show percentages in each group; p values are from Fisher exact tests comparing the proportions during the open versus closed periods.

Questionnaires were returned by 107 (84%) of 128 students. Approximately 100 students (range 99–103, depending on place visited) stated how frequently they would visit public places while school was open, and 46 stated how many times they visited these places during school closure; 45 (98%) of 46 visited >1 place. Fewer students visited shops, places of worship, parks, and playing fields at least 1×/week when school was closed than when open (Figure 1). For other places, frequency of visits did not differ.

Among those who provided information about caregivers, 93 (95%) of 98 reported that >1 adult looked after them during school closure; 49% reported having 2 caregivers (range 1–5). Among caregivers for whom further information was available, 125 (69%) of 182 would have seen the student on a typical school day, 54 (31%) of 173 typically worked outside the home, and 12 (34%) of 35 took time off work to care for the student during school closure.

Among students, 73 provided number of contacts on a typical day during school closure; 35 also provided information for a typical school day, and another 6 only provided information for a typical school day. We therefore conducted unpaired and paired analyses on data from 79 and 35 respondents, respectively. Students who provided contact data were most likely to be in years 7 or 9 but were otherwise similar to those who did not.

**Figure 2:**
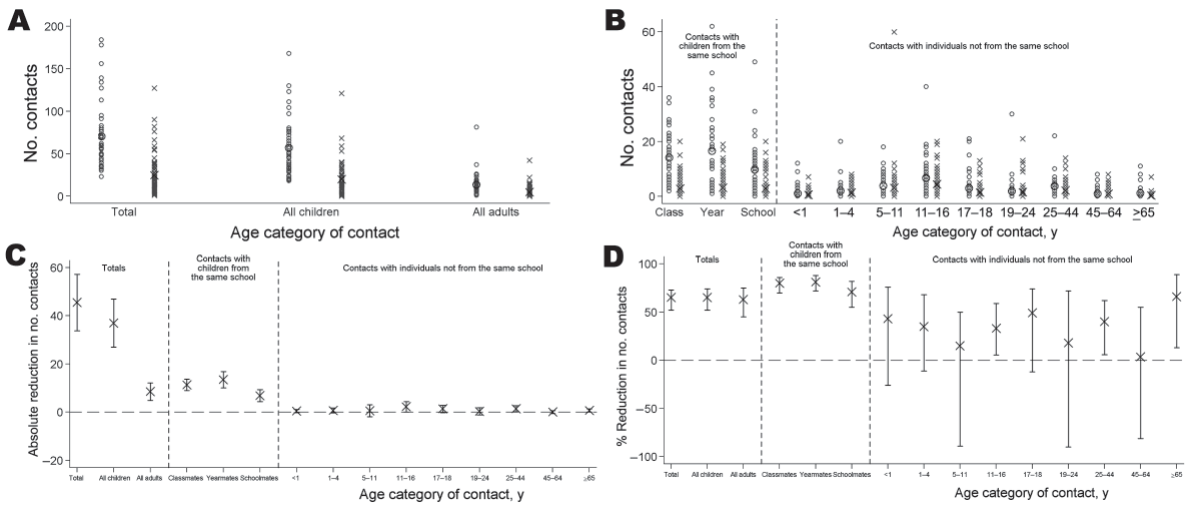
Number of contacts made by students with persons in different categories and the changes associated with school closures. A) total contacts overall and with students and adults; B) contacts with persons in different categories at school and in different age groups outside school; C) absolute reductions in numbers of contacts with persons in different groups associated with school closure; D) relative reductions in numbers of contacts with persons in different groups associated with school closure. In (A) and (B), large black markers indicate the mean number of contacts; small gray markers indicate individual data points; circles indicate data for when the school was open (n = 41), crosses indicate data for when the school was closed (n = 73). In (C) and (D), error bars indicate 95% confidence intervals.

The mean totals of reported contacts were 70.3 (SD 40.8) and 24.8 (SD 22.5) during typical school days and closure, respectively (Figure 2). School closure was therefore associated with a reduction of 45.5 (95% confidence interval [CI] 33.8–57.2) in students’ typical daily number of contacts, a 65% relative reduction (95% bootstrap CI 52%–73%). The corresponding absolute and relative reductions in numbers of contacts with other students were 37.0 (95% CI 27.0–46.9) and 65% (95% bootstrap CI 52%–74%), respectively. The absolute and relative reductions in the numbers of contacts made with adults (including teachers) were 8.5 (95% CI 4.9–12.1) and 63% (95% bootstrap CI 45%–75%), respectively. No apparent change was found for number of contacts with adults outside school (34%, 95% bootstrap CI –6% to 63%).

The greatest reductions in the numbers of contacts were for students from the same school (Figure 2), e.g., ≈80% reduction in numbers of contacts with classmates and yearmates. Absolute reductions in numbers of contacts with persons not attending the school were small; the relative reductions had wide confidence intervals and rarely showed evidence of a genuine reduction (Figure 2). Paired analysis of data for 35 students with information for contacts during both periods produced similar results as unpaired analysis. Among 40 respondents who reported illness during closure and self-assessed whether they consequently contacted fewer persons, 53% stated that their contacts were reduced, 33% stated that they were not, and 15% were unsure.

## Conclusions

Closing this school was associated with a 65% reduction in face-to-face conversational contacts made by secondary school students, primarily because of reductions in contact with students from the same school. Our estimated reductions exceed estimates from analyses of surveillance data for seasonal influenza-like illness in France (24% reduction in child-to-child transmission during school holidays compared with in-school days) (*8*) and a study conducted in Belgium (19% reduction in total contacts made by children and adolescents during Easter holidays) (*9*). Our estimate of a 65% reduction in total contacts is similar to that from a survey at a primary school in Germany, in which students reported 72% fewer contacts on Sundays than on weekdays but in which all classmates were considered contacts (*10*). Consistent with findings of other studies (*11**–**13*), most students visited public places during closure, although certain places were visited less frequently while the school was closed than when open.

Our study has several limitations. Our definition of contact excluded nonconversational contacts (e.g., passengers on public transport), which may enable transmission, and some conversations may not involve close contact. We did not collect data about duration or intensity of contact or whether persons were contacted multiple times. Our use of a typical day does not capture variation in student behavior.

For logistical reasons, a 2–3 week delay occurred between school reopening and completion of questionnaires, providing potential for recall bias and underestimation of numbers of contacts during school closure (although closure was unusual). Prospective data collection was impossible and has limitations, including greater effort required from participants and therefore potentially lower response rates. The data refer to a convenience sample from 1 secondary school during what was often perceived as a mild pandemic and may not be generalizable to other situations (e.g., primary schools, different socioeconomic settings, infections with high case-fatality rates, or different seasons).

Most students provided data only for the closure period, and few did so for a typical school day (probably because of the order of questions). The primary analysis therefore ignored the pairing in the data. Ignoring the pairing would not affect point estimates but would reduce their precision. Paired analysis of 35 students who provided data for both periods produced similar results to the unpaired analysis.

Other issues must also be considered when deciding whether to close schools (*14*). Subject to the limitations described above, reactive school closures may substantially reduce the numbers of contacts made by students and may potentially reduce transmission of infection in some settings.
